# A machine learning–based risk prediction framework for atypical hyperplasia and endometrial cancer in postmenopausal women

**DOI:** 10.1186/s12957-026-04424-1

**Published:** 2026-05-26

**Authors:** Chongjing Li, Jingtao Wang, Yunfeng Zhang, Lening Hu, Yue Wang

**Affiliations:** 1 Department of Gynecology, Henan Provincial People’s Hospital, 7 Weiwu Road, Jinshui District, Zhengzhou, 450003 Henan China; 2 Department of Obstetrics and Gynecology, People’s Hospital of Henan University, Henan Provincial People’s Hospital, Zhengzhou, Henan China; 3 Department of Obstetrics and Gynecology, Zhengzhou University People’s Hospital, Henan Provincial People’s Hospital, Zhengzhou, Henan China

**Keywords:** Endometrial cancer, Atypical hyperplasia, Machine learning, Postmenopausal bleeding, Endometrial thickness, Artificial intelligence

## Abstract

**Background:**

This study aimed to develop and internally validate a machine learning–based model for predicting endometrial malignancy, defined as atypical hyperplasia or endometrial cancer (AH/EC), in postmenopausal women, integrating routinely available clinical, ultrasound, and laboratory features to support individualized diagnostic triage and potentially reduce unnecessary invasive diagnostic procedures in low-risk patients.

**Methods:**

This retrospective, single-center study included 858 postmenopausal women who underwent endometrial histopathological evaluation at Henan Provincial People’s Hospital between February 2022 and September 2025. The cohort was randomly divided into a training set (70%, *n* = 602) and a validation set (30%, *n* = 256). Feature selection was performed in the training cohort using univariate analysis (*P* < 0.001), LASSO regression with the λ₁se criterion, and the Boruta algorithm. Nine supervised machine learning models were developed in the training cohort and evaluated in the validation cohort. Model performance was assessed based on discrimination (area under the receiver operating characteristic curve [AUC], sensitivity, specificity, F1 score), calibration (Brier score, calibration curves), and clinical utility (decision curve analysis). SHAP was applied to interpret the optimal model, and a nomogram was constructed based on the Logistic Regression model.

**Results:**

A total of 155 patients (18.1%) were diagnosed with AH/EC. Six predictors were retained for model development: Endometrial thickness, Postmenopausal bleeding, Presence of blood flow signal, CA19-9, CA125, and Lesion outline regularity. In the validation cohort, the Neural Network model showed the highest AUC (0.840, 95% CI: 0.770–0.909), comparable to Logistic Regression (AUC 0.838, 95% CI: 0.768–0.908), with higher sensitivity (0.739 vs. 0.674) and similar calibration (Brier score 0.099 for both models). Both models showed acceptable validation performance.

**Conclusion:**

A prediction framework based on routinely obtainable clinical, ultrasound, and laboratory variables may support personalized risk assessment in postmenopausal women undergoing diagnostic evaluation for suspected endometrial lesions. Further multicenter external validation and prospective studies are needed to confirm its generalizability and clinical applicability.

**Supplementary Information:**

The online version contains supplementary material available at 10.1186/s12957-026-04424-1.

## Introduction

Endometrial cancer is one of the most common gynecological malignancies [1]. Global cancer statistics for 2022 reported approximately 420,242 new cases and 97,704 deaths worldwide, reflecting the persistent burden of the disease [2]. Epidemiological data suggest a lifetime risk of approximately 3% for developing endometrial cancer, and in some regions, its mortality burden has surpassed that of ovarian cancer, underscoring the long-term impact of this disease on women’s health and public health systems [3]. With the ageing population and the rising prevalence of metabolic risk factors such as obesity and diabetes, the incidence is expected to continue to rise [4, 5]. The prognosis of endometrial cancer is closely associated with the stage at diagnosis. The five-year survival rate for patients with stage I disease ranges from 80% to 90%, whereas for those with stage III and stage IV disease, the rates decline to 50%–65% and 15%–17%, respectively [6]. These findings highlight the significance of early detection and timely intervention in improving clinical outcomes. More than 70% of cases occur in postmenopausal women, with most presenting at an early stage with abnormal uterine bleeding, offering a critical opportunity for early clinical evaluation and risk assessment [1, 7, 8].

In current clinical practice, postmenopausal bleeding, endometrial thickening, and intrauterine space-occupying lesions represent the most prevalent indications for endometrial histological examination. Endometrial thickness (ET), in conjunction with postmenopausal bleeding status, is widely used as an ultrasound parameter for risk stratification. The 2018 guidelines from the American College of Obstetricians and Gynecologists (ACOG) state that in postmenopausal women with bleeding, an endometrial thickness ≤ 4 mm is associated with a low likelihood of endometrial cancer [9]. An endometrial thickness threshold of 4–5 mm is widely used in clinical settings to guide further evaluation in postmenopausal women with abnormal uterine bleeding [10, 11]. Nevertheless, a consensus on the management of endometrial thickening in asymptomatic postmenopausal women remains lacking. The 2024 guideline from the Society of Obstetricians and Gynaecologists of Canada (SOGC) states that in asymptomatic postmenopausal women without bleeding, an endometrial thickness < 11 mm is associated with an extremely low risk of malignancy [12].

Despite these guideline-recommended thresholds, the relationship between ET and malignancy risk is not straightforward. Considerable overlap exists between benign and malignant conditions, and the absolute risk estimates remain relatively modest even beyond conventional cutoffs. Decision-analytic studies further suggest that in women with postmenopausal bleeding and ET > 5 mm, the estimated risk of endometrial cancer is approximately 7.3%, whereas in asymptomatic women with ET > 11 mm, the risk is approximately 6.7% [13]. Moreover, malignancy risk is influenced by age and metabolic risk factors [13]. Previous studies have reported that approximately 20% of women undergoing evaluation for postmenopausal bleeding are diagnosed with endometrial cancer or atypical hyperplasia, whereas the proportion is below 10% among asymptomatic women evaluated solely for endometrial thickening [14]. Collectively, these findings indicate that symptom-driven triage strategies based on fixed ET thresholds remain associated with considerable uncertainty in risk discrimination. Meanwhile, the malignancy risk associated with postmenopausal intrauterine space-occupying lesions has not been well quantified, and clinical triage still relies largely on an integrated assessment of symptoms and sonographic morphology.

Histological examination of the endometrium remains the gold standard for diagnosing malignant endometrial lesions. However, tissue sampling through curettage or hysteroscopy-guided biopsy is invasive and may cause procedure-related complications. Therefore, improving triage efficiency while avoiding unnecessary invasive procedures remains an important clinical challenge. Recent bibliometric evidence indicates that artificial intelligence applications in obstetrics and gynecology are rapidly expanding, particularly in the development of diagnostic prediction models [15]. In this study, histologically confirmed atypical hyperplasia or endometrial cancer (AH/EC) was defined as the primary outcome, and clinical, serological, and ultrasound features were integrated to develop and compare multiple predictive models, including Logistic Regression and several supervised machine learning algorithms, to enable individualized risk stratification in postmenopausal women.

## Methods

### Study population

This retrospective, single-center study included 858 postmenopausal women who underwent endometrial histopathological evaluation at Henan Provincial People’s Hospital between February 2022 and September 2025.

Inclusion criteria were: (1) natural menopause ≥ 12 months with age at menopause > 40 years; (2) diagnostic curettage or hysteroscopy-guided biopsy performed for postmenopausal bleeding (PMB) or abnormal transvaginal ultrasound findings (endometrial thickness [ET] ≥ 4 mm, heterogeneous echogenicity, or intracavitary lesions); (3) definitive histopathological diagnosis; and (4) complete preoperative clinical, ultrasound, and laboratory data.

The exclusion criteria were: (1) iatrogenic menopause; (2) recent hormone replacement therapy; (3) concurrent non-endometrial malignancies; and (4) missing key clinical, imaging, or pathological data.

**Fig. 1:**
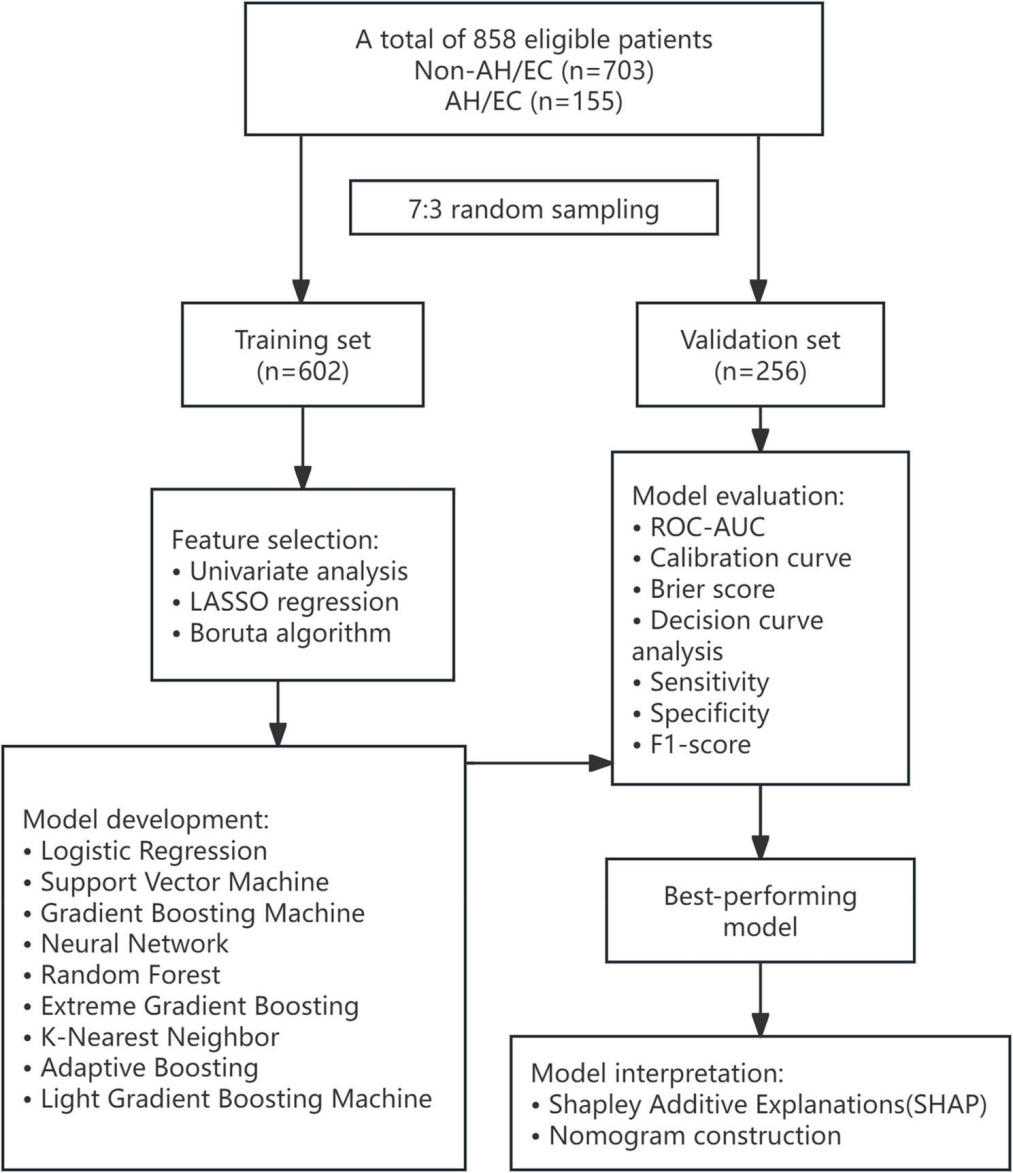
Study population selection and machine learning model development process

The cohort was randomly divided into a training set (*n* = 602) and a validation set (*n* = 256) at a 7:3 ratio. The overall study design and analytical workflow are illustrated in Fig. 1.

### Data collection

Clinical data were retrieved from the electronic medical record system, including demographic and reproductive variables (age, age at menarche, age at menopause, duration of menopause, gravidity, parity, height, weight, and BMI), clinical symptoms (postmenopausal bleeding, vaginal discharge, and abdominal pain), and comorbidities (hypertension, diabetes, obesity, family history of cancer, history of abortion, and history of cesarean delivery). Transvaginal ultrasound parameters were recorded according to the International Endometrial Tumor Analysis (IETA) terminology system and included endometrial thickness (< 5 mm, 5–<10 mm, ≥ 10 mm), endometrial echogenicity (uniform/non-uniform), intracavitary lesion (yes/no), lesion echogenicity, lesion homogeneity, lesion outline regularity, presence of blood flow signal, and intracavitary fluid [16]. For lesion-related variables, “no intracavitary lesion” was treated as a separate category, and these features were modeled as unordered categorical variables. Preoperative laboratory variables included CA125, CA19-9, fasting blood glucose, serum uric acid, hemoglobin, fibrinogen, and D-dimer. CA125 and CA19-9 were categorized as ≤ 35 U/mL or > 35 U/mL according to institutional reference ranges.

### Pathological evaluation

All endometrial specimens were independently reviewed by two experienced gynecologic pathologists. Diagnoses were made according to the WHO Classification of Tumours of the Female Genital Tract. Based on the final histopathological diagnosis, patients were categorized into the AH/EC group (atypical hyperplasia and endometrial cancer) and the non-AH/EC group (atrophic endometrium, endometrial polyps, endometrial hyperplasia without atypia, and other benign lesions).

### Cohort partitioning and feature selection

Multiple imputation was used to address limited missing data and reduce potential bias. After imputation, participants were randomly allocated to a training set and a validation set at a 7:3 ratio. In the training cohort, univariate analysis was first performed to screen candidate variables, and variables with a P value < 0.001 were retained for further feature selection. Subsequently, least absolute shrinkage and selection operator (LASSO) regression was conducted, with the penalty parameter determined using the λ₁se criterion through cross-validation. The Boruta algorithm was also applied for feature selection. Predictors consistently retained by univariate analysis, LASSO regression, and Boruta were selected for subsequent model development.

### Machine learning models and evaluation

Based on the selected predictors, nine supervised machine learning models were developed to estimate the probability of endometrial malignancy, including Logistic Regression (LR), Support Vector Machine (SVM), Gradient Boosting Machine (GBM), Neural Network (NN), Random Forest (RF), Extreme Gradient Boosting (XGBoost), K-Nearest Neighbor (KNN), Adaptive Boosting (AdaBoost), and Light Gradient Boosting Machine (LightGBM). Hyperparameters were optimized in the training set using grid search with k-fold cross-validation, based on the mean cross-validated area under the receiver operating characteristic curve (AUC). Each model was trained on the full training set and evaluated in the validation set. Model performance was assessed using AUC, sensitivity, specificity, F1 score, calibration curves, Brier score, and decision curve analysis.

### Model interpretability

To enhance model interpretability, model-specific explanation approaches were applied according to the algorithm type. For the machine learning algorithms, SHapley Additive exPlanations (SHAP) were applied to quantify the contribution of each feature to the predicted probability and to evaluate their relative importance. Based on SHAP values, feature importance plots, SHAP summary (beeswarm) plots, and individual-level waterfall plots were generated to illustrate the magnitude and direction of feature effects at both the population and individual levels.

For the Logistic Regression (LR) model, odds ratios (ORs) with corresponding 95% confidence intervals (CIs) were derived from the regression coefficients to quantify the independent associations between each predictor and the outcome. A nomogram was subsequently constructed based on this LR model to facilitate individualized risk estimation and clinical interpretation.

### Statistical analysis

All statistical analyses and model development were conducted using R software (version 4.4.1). Continuous variables were presented as mean ± standard deviation (SD) for normally distributed data or median (interquartile range, IQR) for non-normally distributed data. Between-group comparisons were conducted using the independent-samples t-test or the Mann–Whitney U test, as appropriate. Categorical variables were expressed as counts and percentages [n (%)] and were compared using the chi-square test or Fisher’s exact test. All statistical tests were two-sided, and a *P* value < 0.05 was considered indicative of statistical significance.

## Results

### Patient characteristics

A total of 858 postmenopausal women were included in the analysis, of whom 155 (18.1%) were diagnosed with AH/EC and 703 (81.9%) with non-AH/EC lesions. The baseline characteristics of the study population are summarized in Table 1. Compared with the non-AH/EC group, patients with AH/EC were older and had a longer duration of menopause (both *P* < 0.05). No statistically significant differences were observed in age at menarche, age at menopause, gravidity, parity, height, weight, or BMI (all *P* > 0.05). Postmenopausal bleeding and vaginal discharge were more frequent in the AH/EC group (both *P* < 0.05), whereas abdominal pain did not differ significantly between groups (*P* > 0.05). Hypertension and diabetes were more frequent in the AH/EC group (both *P* ≤ 0.01), whereas other comorbidities were comparable between groups. Regarding ultrasound features, patients with AH/EC more frequently presented with endometrial thickness ≥ 10 mm (65.2% vs. 22.3%, *P* < 0.001), non-uniform endometrial echogenicity, presence of blood flow signal, and intracavitary lesions (all *P* < 0.001). Among patients with intracavitary lesions, irregular lesion outline and non-uniform lesion homogeneity were significantly more prevalent in the AH/EC group (both *P* < 0.001). The proportion of intracavitary fluid did not differ significantly between groups. Laboratory findings demonstrated significantly higher proportions of elevated CA125 and CA19-9 levels in the AH/EC group (both *P* < 0.001). In addition, fasting blood glucose, serum uric acid, and fibrinogen levels were higher, whereas hemoglobin levels were lower in the AH/EC group (all *P* < 0.05). No significant difference was observed in D-dimer levels.

**Table 1:** Baseline characteristics of patients

Characteristic	Overall (*n* = 858)	Non-AH/EC (*n* = 703)	AH/EC (*n* = 155)	*P* value
Age (year)	59.50 ± 7.41	59.17 ± 7.29	60.96 ± 7.78	0.007
Age at menarche (years)	14.23 ± 1.70	14.28 ± 1.71	13.99 ± 1.66	0.054
Age at menopause (years)	50.38 ± 3.44	50.35 ± 3.33	50.55 ± 3.90	0.513
Duration of menopause (years)	9.11 ± 7.55	8.83 ± 7.49	10.41 ± 7.72	0.018
Gravidity	2.82 ± 1.57	2.85 ± 1.62	2.72 ± 1.32	0.35
Parity	2.05 ± 1.03	2.04 ± 1.03	2.07 ± 1.03	0.744
Height (cm)	158.99 ± 5.53	159.02 ± 5.50	158.84 ± 5.71	0.708
Weight (kg)	63.53 ± 9.70	63.36 ± 9.85	64.33 ± 8.96	0.26
BMI (kg/m²)	25.12 ± 3.55	25.04 ± 3.60	25.49 ± 3.30	0.152
Postmenopausal bleeding (%)				< 0.001
No	413 (48.1)	379 (53.9)	34 (21.9)	
Yes	445 (51.9)	324 (46.1)	121 (78.1)	
Vaginal discharge (%)				0.025
No	797 (92.9)	660 (93.9)	137 (88.4)	
Yes	61 ( 7.1)	43 ( 6.1)	18 (11.6)	
Abdominal pain (%)				0.099
No	699 (81.5)	565 (80.4)	134 (86.5)	
Yes	159 (18.5)	138 (19.6)	21 (13.5)	
Endometrial thickness (%)				< 0.001
< 5 mm	243 (28.3)	238 (33.9)	5 ( 3.2)	
5–<10 mm	357 (41.6)	308 (43.8)	49 (31.6)	
≥ 10 mm	258 (30.1)	157 (22.3)	101 (65.2)	
Endometrial echogenicity (%)				< 0.001
Uniform	249 (29.0)	231 (32.9)	18 (11.6)	
Non-uniform	609 (71.0)	472 (67.1)	137 (88.4)	
Intracavitary lesion (%)				< 0.001
No	468 (54.5)	406 (57.8)	62 (40.0)	
Yes	390 (45.5)	297 (42.2)	93 (60.0)	
Lesion echogenicity (%)				< 0.001
No intracavitary lesion	468 (54.5)	406 (57.8)	62 (40.0)	
Hyperechogenic	314 (36.6)	247 (35.1)	67 (43.2)	
Hypoechogenic	61 ( 7.1)	42 ( 6.0)	19 (12.3)	
Isoechogenic	15 ( 1.7)	8 ( 1.1)	7 ( 4.5)	
Lesion homogeneity (%)				< 0.001
No intracavitary lesion	468 (54.5)	406 (57.8)	62 (40.0)	
Uniform	90 (10.5)	77 (11.0)	13 ( 8.4)	
Non-uniform	300 (35.0)	220 (31.3)	80 (51.6)	
Lesion outline regularity (%)				< 0.001
No intracavitary lesion	468 (54.5)	406 (57.8)	62 (40.0)	
Regular	252 (29.4)	220 (31.3)	32 (20.6)	
Irregular	138 (16.1)	77 (11.0)	61 (39.4)	
Presence of blood flow signal (%)				< 0.001
No	642 (74.8)	577 (82.1)	65 (41.9)	
Yes	216 (25.2)	126 (17.9)	90 (58.1)	
Intracavitary fluid (%)				1
No	650 (75.8)	533 (75.8)	117 (75.5)	
Yes	208 (24.2)	170 (24.2)	38 (24.5)	
Hypertension (%)				0.006
No	513 (59.8)	436 (62.0)	77 (49.7)	
Yes	345 (40.2)	267 (38.0)	78 (50.3)	
Diabetes (%)				< 0.001
No	637 (74.2)	544 (77.4)	93 (60.0)	
Yes	221 (25.8)	159 (22.6)	62 (40.0)	
Obesity (%)				0.562
No	687 (80.1)	566 (80.5)	121 (78.1)	
Yes	171 (19.9)	137 (19.5)	34 (21.9)	
Family history of cancer (%)				0.207
No	677 (78.9)	561 (79.8)	116 (74.8)	
Yes	181 (21.1)	142 (20.2)	39 (25.2)	
History of abortion (%)				0.884
No	522 (60.8)	429 (61.0)	93 (60.0)	
Yes	336 (39.2)	274 (39.0)	62 (40.0)	
History of cesarean delivery (%)				0.084
No	780 (90.9)	633 (90.0)	147 (94.8)	
Yes	78 ( 9.1)	70 (10.0)	8 ( 5.2)	
CA125 (%)				< 0.001
≤ 35 U/mL	813 (94.8)	692 (98.4)	121 (78.1)	
> 35 U/mL	45 ( 5.2)	11 ( 1.6)	34 (21.9)	
CA19-9 (%)				< 0.001
≤ 35 U/mL	797 (92.9)	682 (97.0)	115 (74.2)	
> 35 U/mL	61 ( 7.1)	21 ( 3.0)	40 (25.8)	
Fasting blood glucose (mmol/L)	5.51 ± 1.54	5.43 ± 1.51	5.85 ± 1.62	0.002
Serum uric acid (µmol/L)	277.21 ± 70.73	271.79 ± 67.58	301.79 ± 79.25	< 0.001
Hemoglobin (g/L)	129.84 ± 11.96	130.22 ± 11.56	128.07 ± 13.50	0.042
Fibrinogen (g/L)	2.77 ± 0.60	2.73 ± 0.57	2.94 ± 0.74	< 0.001
D-dimer (µg/mL)	0.36 ± 0.50	0.35 ± 0.40	0.42 ± 0.79	0.112

Values are presented as mean ± SD or n (%)

*P* values were calculated using t test or Mann–Whitney U test for continuous variables and χ² or Fisher’s exact test for categorical variables

*AH/EC* Atypical endometrial hyperplasia/endometrial cancer, *BMI* Body mass index, *CA125* Carbohydrate antigen 125, *CA19-9* Carbohydrate antigen 19 − 9. CA125 and CA19-9 were categorized according to the institutional upper reference limit (35 U/mL)

### Model development and comparison

**Fig. 2:**
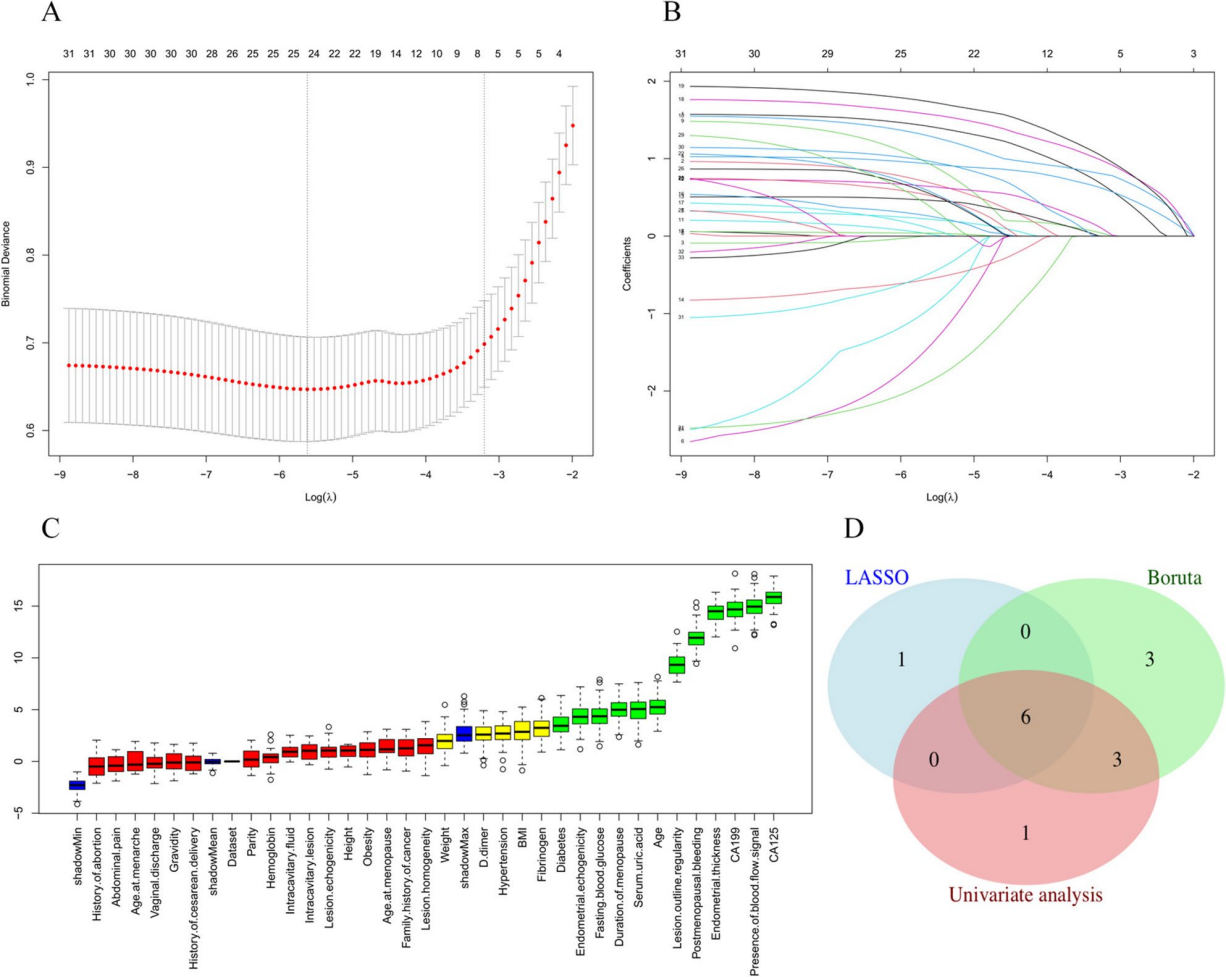
Feature selection procedures and identification of candidate predictors. **A** Ten-fold cross-validation curve for LASSO regression showing the optimal penalty parameter (λ). The vertical dashed lines indicate λ_min and λ_1se. **B** LASSO coefficient trajectories plotted against log(λ), illustrating coefficient shrinkage as the penalty increases. **C** Variable importance ranking generated by the Boruta algorithm. **D** Venn diagram showing the overlap of predictors selected by univariate analysis, LASSO regression, and Boruta algorithm. Six variables were identified at the intersection and subsequently included in multivariable modeling

**Fig. 3:**
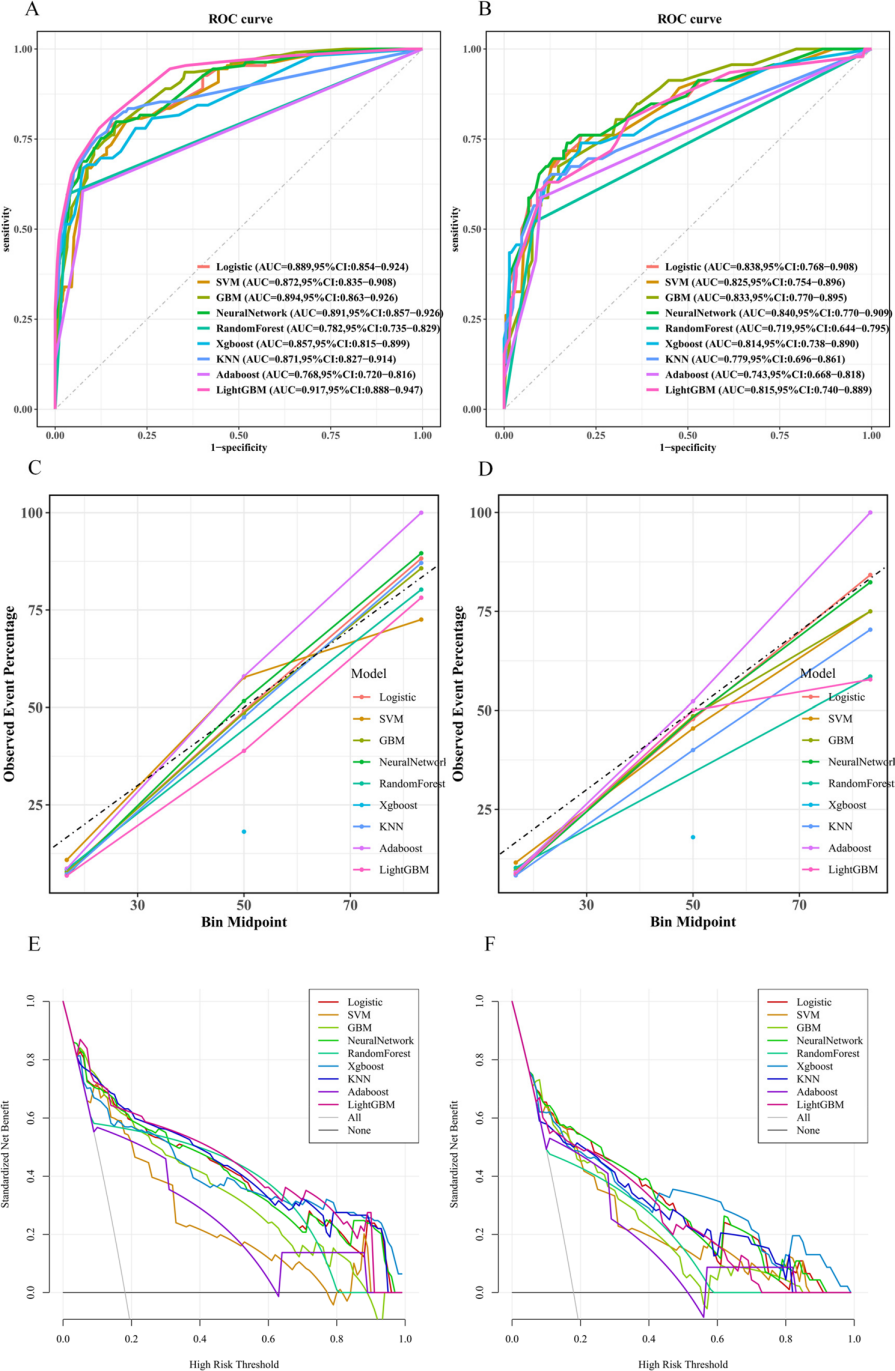
Receiver operating characteristic (ROC) curves, calibration plots, and decision curve analyses of machine learning models in the training and validation sets. **A** ROC curves of the nine machine learning models in the training set. **B** ROC curves in the validation set. **C** Calibration curves in the training set. **D** Calibration curves in the validation set. **E** Decision curve analysis (DCA) in the training set. **F** Decision curve analysis in the validation set

The baseline characteristics of the training and internal validation sets are presented in Supplementary Table S1. No significant differences were observed between the two datasets across demographic, clinical, ultrasound, and laboratory variables (all *P* > 0.05), suggesting that the random partitioning maintained good comparability between the two cohorts. In the training set, variables with *P* < 0.001 in univariate analysis were retained for feature selection (Supplementary Table S2). LASSO and Boruta identified seven and twelve candidate variables, respectively. Six predictors were ultimately retained at the intersection of the three methods (Fig. 2) and used for model construction. Nine supervised machine learning models were developed and evaluated: Logistic Regression, SVM, GBM, Neural Network, Random Forest, XGBoost, KNN, AdaBoost, and LightGBM. The receiver operating characteristic curves, calibration curves, and decision curve analyses are presented in Fig. 3, and the detailed performance metrics are summarized in Tables 2 and 3.

In the training set, AUC values ranged from 0.768 to 0.917 across models. LightGBM achieved the highest AUC (0.917, 95% CI: 0.888–0.947), followed by GBM (0.894, 95% CI: 0.863–0.926), Neural Network (0.891, 95% CI: 0.857–0.926), and Logistic Regression (0.889, 95% CI: 0.854–0.924). The Neural Network achieved a sensitivity of 0.798 and specificity of 0.834 in the training set, with a Brier score of 0.085. In the validation set, the Neural Network achieved an AUC of 0.840 (95% CI: 0.770–0.909), with a sensitivity of 0.739, specificity of 0.829, and a Brier score of 0.099. Logistic Regression achieved an AUC of 0.838 (95% CI: 0.768–0.908) and a specificity of 0.886 in the validation set. Boosting-based models, including LightGBM and GBM, showed larger decreases in AUC from the training to the validation set compared with Neural Network and Logistic Regression, whereas Random Forest and AdaBoost demonstrated relatively lower AUC values in both datasets. Given its performance in the validation set, the Neural Network was selected for subsequent model interpretation. Logistic Regression was retained for comparison and nomogram construction due to its interpretability.

**Table 2:** Predictive performance of machine learning algorithms in the training set

Model	Sensitivity	Specificity	F1-score	Accuracy	Brier score	AUC (95% CI)
Logistic Regression	0.780	0.856	0.642	0.842	0.083	0.889(0.854–0.924)
SVM	0.807	0.801	0.597	0.802	0.105	0.872(0.835–0.908)
GBM	0.725	0.895	0.658	0.864	0.089	0.894(0.863–0.926)
Neural Network	0.798	0.834	0.626	0.827	0.085	0.891(0.857–0.926)
Random Forest	0.596	0.968	0.684	0.900	0.100	0.782(0.735–0.829)
XGBoost	0.670	0.923	0.664	0.877	0.247	0.857(0.815–0.899)
KNN	0.807	0.844	0.642	0.837	0.081	0.871(0.827–0.914)
AdaBoost	0.606	0.925	0.623	0.867	0.112	0.768(0.720–0.816)
LightGBM	0.780	0.880	0.672	0.862	0.078	0.917(0.888–0.947)

**Table 3:** Predictive performance of machine learning algorithms in the validation set

Model	Sensitivity	Specificity	F1-score	Accuracy	Brier score	AUC (95% CI)
Logistic Regression	0.674	0.886	0.614	0.848	0.099	0.838(0.768–0.908)
SVM	0.696	0.857	0.593	0.828	0.111	0.825(0.754–0.896)
GBM	0.674	0.852	0.574	0.820	0.112	0.833(0.770–0.895)
Neural Network	0.739	0.829	0.586	0.812	0.099	0.840(0.770–0.909)
Random Forest	0.522	0.919	0.552	0.848	0.152	0.719(0.644–0.795)
XGBoost	0.739	0.790	0.548	0.781	0.247	0.814(0.738–0.890)
KNN	0.652	0.871	0.583	0.832	0.114	0.779(0.696–0.861)
AdaBoost	0.587	0.900	0.574	0.844	0.126	0.743(0.668–0.818)
LightGBM	0.609	0.910	0.602	0.855	0.117	0.815(0.740–0.889)

### Model explainability

**Fig. 4:**
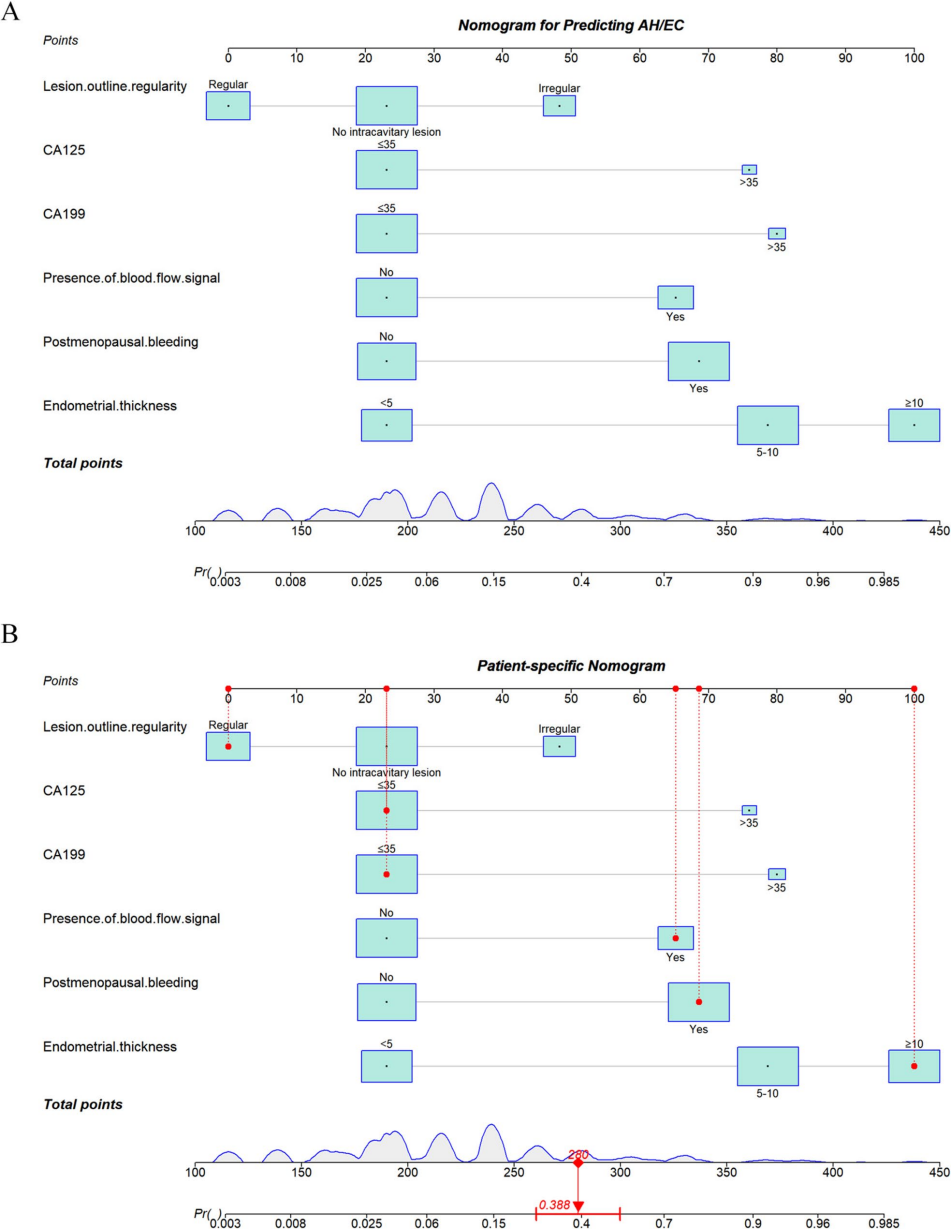
Nomogram derived from the Logistic Regression model and individualized risk estimation. **A** Construction of the nomogram incorporating six independent predictors. **B** Illustration of patient-specific risk calculation based on total points

Based on the six predictors selected through the feature selection procedure, the Logistic Regression model was constructed (Table 4), and a corresponding nomogram was developed (Fig. 4A). Each predictor corresponds to a point value, and the total score corresponds to the predicted probability of AH/EC. Figure 4B illustrates an example of patient-specific prediction using the nomogram, in which the red lines indicate the point assignment for each predictor and the summed total points, corresponding to an estimated AH/EC probability of approximately 0.39.

**Fig. 5:**
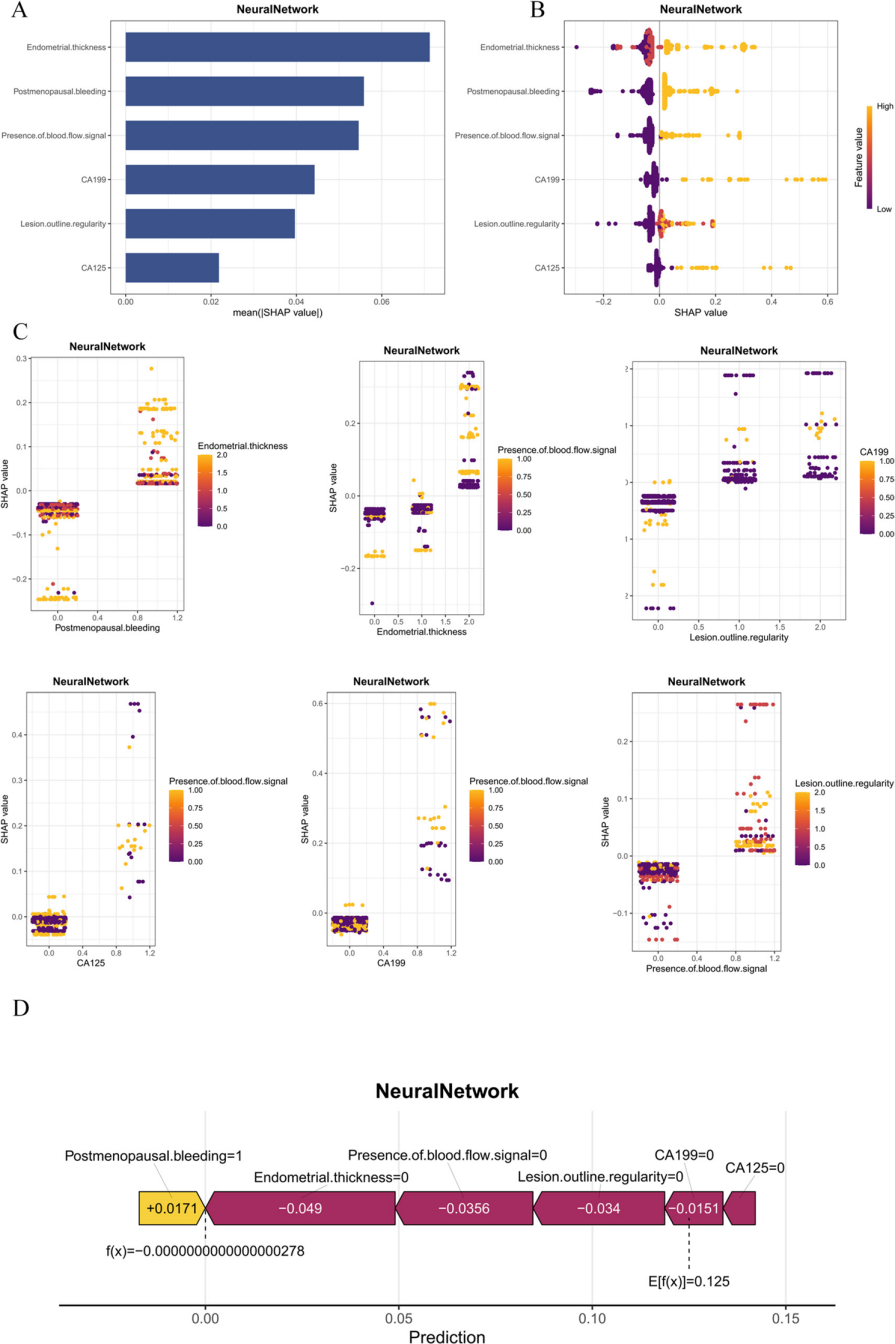
SHAP analysis for interpretation of the Neural Network model. **A** Feature importance based on mean absolute SHAP values. **B** SHAP summary plot displaying the distribution and direction of feature effects. **C** SHAP dependence plots illustrating the marginal effect of key predictors. **D** SHAP force plot showing individual-level prediction decomposition

SHapley Additive exPlanations (SHAP) were applied to the Neural Network model to quantify the contribution of each predictor to the predicted probability of AH/EC. The SHAP summary (beeswarm) plot (Fig. 5B) showed that Endometrial thickness had the highest mean absolute SHAP value, followed by Postmenopausal bleeding, Presence of blood flow signal, CA19-9, Lesion outline regularity, and CA125. SHAP dependence plots (Fig. 5C) depict the relationship between selected feature values and their corresponding SHAP contributions. Representative SHAP waterfall plots (Fig. 5D) demonstrate the additive contribution of individual predictors to the predicted probability in a single case.

**Table 4:** Multivariable logistic regression analysis of independent predictors for AH/EC

Variable	Beta	OR (95% CI)	*P* value
Endometrial thickness			
< 5 mm		ref	
5–<10 mm	1.793	6.01 (2.16–16.72)	<0.001
≥ 10 mm	2.481	11.95 (4.27–33.42)	<0.001
Postmenopausal bleeding			
No		ref	
Yes	1.468	4.34 (2.41–7.81)	<0.001
Presence of blood flow signal			
No		ref	
Yes	1.359	3.89 (1.95–7.77)	<0.001
CA19-9 (%)			
≤ 35 U/mL		ref	
> 35 U/mL	1.835	6.27 (2.50–15.72)	<0.001
CA125 (%)			
≤ 35 U/mL		ref	
> 35 U/mL	1.705	5.51 (1.89–16.03)	0.002
Lesion outline regularity			
No intracavitary lesion		ref	
Regular	-0.746	0.47 (0.22–1.04)	0.062
Irregular	0.812	2.25 (1.08–4.70)	0.030

## Discussion

A machine learning–based predictive framework for AH/EC in postmenopausal women was developed and internally validated in this study. Using six routinely available clinical, ultrasound, and laboratory predictors, nine supervised machine learning models were constructed and compared. In the validation cohort, the Neural Network model achieved the highest AUC of 0.840, which was comparable to Logistic Regression. The Neural Network model showed relatively higher sensitivity, whereas Logistic Regression showed higher specificity and better interpretability. These findings suggest that both models may provide complementary value for individualized risk assessment in postmenopausal women undergoing diagnostic evaluation.

A multistep feature selection strategy incorporating univariate analysis, LASSO regression, and the Boruta algorithm identified six predictors: Endometrial thickness, Postmenopausal bleeding, Presence of blood flow signal, CA19-9, CA125, and Lesion outline regularity. These variables are clinically accessible and reflect different dimensions of malignancy risk, including structural endometrial changes, symptom status, vascular characteristics, lesion morphology, and serum tumor marker alterations. Multivariable logistic regression confirmed their independent associations with AH/EC, while SHAP analysis further demonstrated their relative contributions in the Neural Network model.

Endometrial thickness was the most important predictor in both regression and SHAP analyses. This finding is consistent with previous evidence showing that increased endometrial thickness is associated with a higher probability of endometrial malignancy, whereas relatively thin endometrium has a high negative predictive value for excluding endometrial cancer in postmenopausal women [9, 13]. However, the diagnostic value of ET depends strongly on clinical context, and fixed thresholds may be insufficient for accurate risk stratification, particularly in asymptomatic women or in tumors that may occur without marked endometrial thickening [12, 14]. Recent evidence from women with abnormal uterine bleeding also suggests that endometrial thickness measured by transvaginal ultrasonography is associated with histopathological findings but remains insufficient as a definitive diagnostic marker [17]. Therefore, ET should be interpreted together with clinical, sonographic, and laboratory features rather than used as a standalone decision criterion.

Postmenopausal bleeding was also an important independent predictor of AH/EC. Although PMB is the most common presenting symptom of endometrial cancer, only a minority of women with PMB are ultimately diagnosed with malignancy [18]. Thus, PMB is better regarded as a triage indicator rather than a diagnostic marker. In the present model, the combination of PMB and ET appeared to improve risk discrimination, supporting the clinical rationale that symptom status modifies the significance of endometrial thickening in postmenopausal women.

The presence of blood flow signal was independently associated with AH/EC and showed a positive contribution in the Neural Network model. This finding is biologically plausible, as malignant endometrial lesions are often accompanied by increased angiogenesis and abnormal vascular patterns [19, 20]. Compared with structural parameters alone, Doppler vascular information may provide additional value by reflecting the biological activity of the lesion, especially when interpreted in combination with endometrial thickness and lesion morphology.

Lesion outline regularity was another relevant ultrasound feature in this study. Irregular lesion outline was associated with a higher risk of AH/EC, whereas regular outline did not show a significant independent association. This supports previous observations that malignant or premalignant endometrial lesions are more likely to present with irregular morphology or architectural distortion on ultrasound [21, 22]. Incorporating lesion morphology using standardized IETA terminology may therefore improve the consistency and clinical interpretability of ultrasound-based risk assessment.

CA125 and CA19-9 were also retained in the final model. Although their overall positivity rates were relatively low, elevated levels were more common in the AH/EC group than in the non-AH/EC group. In both regression and SHAP analyses, these markers contributed positively to predicted risk, but their importance was lower than that of ET and PMB. These findings suggest that CA125 and CA19-9 may serve as supplementary risk modifiers rather than primary diagnostic indicators. Previous studies have shown that CA125 may be associated with tumor stage, myometrial invasion, lymphovascular space invasion, and lymph node metastasis in endometrial cancer [23, 24]. CA19-9 may also be elevated in a subset of patients, although its diagnostic sensitivity and specificity remain limited [23]. Because these markers can be affected by benign gynecologic or inflammatory conditions, their clinical value should be interpreted cautiously and integrated with imaging and symptom-based information rather than used independently for screening or diagnosis.

Several previous studies have explored machine learning methods for endometrial cancer detection using metabolomics, fluorescence spectroscopy, volatile organic compounds, or blood-based indices [25–27]. Although these approaches have shown promising performance, many require specialized platforms or high-dimensional assays, which may limit routine clinical implementation. In contrast, the present study used variables that are readily obtainable in standard gynecologic evaluation, including clinical symptoms, transvaginal ultrasound findings, and common serum markers. This may enhance the feasibility of applying the model in real-world diagnostic triage.

Despite these strengths, several limitations should be acknowledged. First, this was a retrospective study conducted at a single tertiary center, which may introduce selection bias and limit the generalizability of the findings. Second, the model was internally validated only, and external validation in independent multicenter cohorts is still required. Third, ultrasound assessment is subject to inter-operator variability, which may affect the reproducibility of imaging features and the consistency of model application across different clinical settings. Fourth, although CA125 and CA19-9 were identified as independent predictors, their overall sensitivity is relatively low, and their clinical value may be limited when used in isolation. Finally, the clinical impact of using this prediction framework to guide biopsy or hysteroscopy decisions remains to be assessed prospectively. Future studies should focus on external validation, prospective evaluation, standardization of ultrasound assessment, and integration of the model into practical diagnostic workflows.

## Conclusion

This study developed and internally validated a predictive model for assessing the risk of endometrial malignancy (AH/EC) in postmenopausal women undergoing clinical evaluation. Among nine evaluated algorithms, the Neural Network and Logistic Regression models demonstrated favorable discrimination and calibration in the internal validation set. This model may serve as a decision-support tool to assist clinicians in risk stratification and to optimize biopsy selection in postmenopausal women undergoing diagnostic evaluation. Further multicenter external validation and prospective studies are needed to confirm its broader clinical applicability.

## Supplementary Information

Supplementary Material 1.

## Data Availability

No datasets were generated or analysed during the current study.
