# Supplementary material for: A machine learning–based risk prediction framework for atypical hyperplasia and endometrial cancer in postmenopausal women
Source: World J Surg Oncol. 2026 May 26;24:309. doi: 10.1186/s12957-026-04424-1 (PMC13397630; doi:10.1186/s12957-026-04424-1)
Supplement: Supplementary file 1 — Supplementary Material 1. [file 12957_2026_4424_MOESM1_ESM.docx]

**Table S1.** Baseline characteristics of the training and validation cohorts

| Characteristic | validation (n=256) | Training (n=602) | P value |
| --- | --- | --- | --- |
| Age (year) | 59.40±7.58 | 59.54±7.34 | 0.798 |
| Age at menarche (years) | 14.36±1.72 | 14.17±1.69 | 0.135 |
| Age at menopause (years) | 50.49±3.39 | 50.34±3.46 | 0.55 |
| Duration of menopause (years) | 8.91±7.82 | 9.20±7.44 | 0.601 |
| Gravidity | 2.80±1.56 | 2.83±1.58 | 0.825 |
| Parity | 2.05±1.02 | 2.04±1.03 | 0.881 |
| Height (cm) | 159.08±5.58 | 158.95±5.52 | 0.749 |
| Weight (kg) | 63.24±10.28 | 63.65±9.45 | 0.571 |
| BMI (kg/m²) | 24.99±3.73 | 25.18±3.48 | 0.471 |
| Postmenopausal bleeding (%) |  |  | 1 |
| No | 123 (48.05) | 290 (48.17) |  |
| Yes | 133 (51.95) | 312 (51.83) |  |
| Vaginal discharge (%) |  |  | 1 |
| No | 238 (92.97) | 559 (92.86) |  |
| Yes | 18 ( 7.03) | 43 ( 7.14) |  |
| Abdominal pain (%) |  |  | 0.436 |
| No | 204 (79.69) | 495 (82.23) |  |
| Yes | 52 (20.31) | 107 (17.77) |  |
| Endometrial thickness (%) |  |  | 0.691 |
| <5 mm | 69 (26.95) | 174 (28.90) |  |
| 5–<10 mm | 105 (41.02) | 252 (41.86) |  |
| ≥10 mm | 82 (32.03) | 176 (29.24) |  |
| Endometrial echogenicity (%) |  |  | 0.431 |
| Uniform | 69 (26.95) | 180 (29.90) |  |
| Non-uniform | 187 (73.05) | 422 (70.10) |  |
| Intracavitary lesion (%) |  |  | 0.535 |
| No | 135 (52.73) | 333 (55.32) |  |
| Yes | 121 (47.27) | 269 (44.68) |  |
| Lesion echogenicity (%) |  |  | 0.891 |
| No intracavitary lesion | 135 (52.73) | 333 (55.32) |  |
| Hyperechogenic | 98 (38.28) | 216 (35.88) |  |
| Hypoechogenic | 19 ( 7.42) | 42 ( 6.98) |  |
| Isoechogenic | 4 ( 1.56) | 11 ( 1.83) |  |
| Lesion homogeneity (%) |  |  | 0.755 |
| No intracavitary lesion | 135 (52.73) | 333 (55.32) |  |
| Uniform | 29 (11.33) | 61 (10.13) |  |
| Non-uniform | 92 (35.94) | 208 (34.55) |  |
| Lesion outline regularity (%) |  |  | 0.757 |
| No intracavitary lesion | 135 (52.73) | 333 (55.32) |  |
| Regular | 77 (30.08) | 175 (29.07) |  |
| Irregular | 44 (17.19) | 94 (15.61) |  |
| Presence of blood flow signal (%) |  |  | 0.612 |

**Table S1.** (continued)

| Characteristic | validation (n=256) | Training (n=602) | P value |
| --- | --- | --- | --- |
| No | 195 (76.17) | 447 (74.25) |  |
| Yes | 61 (23.83) | 155 (25.75) |  |
| Intracavitary fluid (%) |  |  | 0.671 |
| No | 191 (74.61) | 459 (76.25) |  |
| Yes | 65 (25.39) | 143 (23.75) |  |
| Hypertension (%) |  |  | 0.697 |
| No | 150 (58.59) | 363 (60.30) |  |
| Yes | 106 (41.41) | 239 (39.70) |  |
| Diabetes (%) |  |  | 0.353 |
| No | 196 (76.56) | 441 (73.26) |  |
| Yes | 60 (23.44) | 161 (26.74) |  |
| Obesity (%) |  |  | 0.776 |
| No | 207 (80.86) | 480 (79.73) |  |
| Yes | 49 (19.14) | 122 (20.27) |  |
| Family history of cancer (%) |  |  | 0.17 |
| No | 194 (75.78) | 483 (80.23) |  |
| Yes | 62 (24.22) | 119 (19.77) |  |
| History of abortion (%) |  |  | 0.674 |
| No | 159 (62.11) | 363 (60.30) |  |
| Yes | 97 (37.89) | 239 (39.70) |  |
| History of cesarean delivery (%) |  |  | 0.472 |
| No | 236 (92.19) | 544 (90.37) |  |
| Yes | 20 ( 7.81) | 58 ( 9.63) |  |
| CA125 (%) |  |  | 0.98 |
| ≤35 U/mL | 242 (94.53) | 571 (94.85) |  |
| >35 U/mL | 14 ( 5.47) | 31 ( 5.15) |  |
| CA19-9 (%) |  |  | 0.839 |
| ≤35 U/mL | 239 (93.36) | 558 (92.69) |  |
| >35 U/mL | 17 ( 6.64) | 44 ( 7.31) |  |
| Fasting blood glucose (mmol/L) | 5.48±1.43 | 5.52±1.59 | 0.691 |
| Serum uric acid (μmol/L) | 275.31±73.22 | 278.01±69.69 | 0.609 |
| Hemoglobin (g/L) | 129.56±10.88 | 129.95±12.40 | 0.663 |
| Fibrinogen (g/L) | 2.76±0.61 | 2.77±0.60 | 0.825 |
| D-dimer (μg/ml) | 0.40±0.70 | 0.35±0.38 | 0.204 |

**Table S2.** Univariate comparison of baseline characteristics between Non-AH/EC and AH/EC patients in the training cohort

| Characteristic | Non-AH/EC  (n=493) | AH/EC  (n=109) | P value |
| --- | --- | --- | --- |
| Postmenopausal bleeding (%) |  |  | <0.001 |
| Yes | 226 (45.8) | 86 (78.9) |  |
| No | 267 (54.2) | 23 (21.1) |  |

**Table S2.** (continued)

| Characteristic | Non-AH/EC  (n=493) | AH/EC  (n=109) | P value |
| --- | --- | --- | --- |
| Vaginal discharge (%) |  |  | 0.127 |
| Yes | 31 (6.3) | 12 (11.0) |  |
| No | 462 (93.7) | 97 (89.0) |  |
| Abdominal pain (%) |  |  | 0.177 |
| Yes | 93 (18.9) | 14 (12.8) |  |
| No | 400 (81.1) | 95 (87.2) |  |
| Endometrial thickness (%) |  |  | <0.001 |
| <5 mm | 169 (34.3) | 5 (4.6) |  |
| 5–<10 mm | 215 (43.6) | 37 (33.9) |  |
| ≥10 mm | 109 (22.1) | 67 (61.5) |  |
| Endometrial echogenicity (%) |  |  | <0.001 |
| Non-uniform | 327 (66.3) | 95 (87.2) |  |
| Uniform | 166 (33.7) | 14 (12.8) |  |
| Intracavitary lesion (%) |  |  | 0.003 |
| Yes | 206 (41.8) | 63 (57.8) |  |
| No | 287 (58.2) | 46 (42.2) |  |
| Lesion echogenicity (%) |  |  | 0.003 |
| No intracavitary lesion | 287 (58.2) | 46 (42.2) |  |
| Hyperechogenic | 171 (34.7) | 45 (41.3) |  |
| Hypoechogenic | 28 (5.7) | 14 (12.8) |  |
| Isoechogenic | 7 (1.4) | 4 (3.7) |  |
| Lesion homogeneity (%) |  |  | <0.001 |
| No intracavitary lesion | 287 (58.2) | 46 (42.2) |  |
| Uniform | 57 (11.6) | 4 (3.7) |  |
| Non-uniform | 149 (30.2) | 59 (54.1) |  |
| Lesion outline regularity (%) |  |  | <0.001 |
| No intracavitary lesion | 287 (58.2) | 46 (42.2) |  |
| Regular | 153 (31.0) | 22 (20.2) |  |
| Irregular | 53 (10.8) | 41 (37.6) |  |
| Presence of blood flow signal (%) |  |  | <0.001 |
| Yes | 91 (18.5) | 64 (58.7) |  |
| No | 402 (81.5) | 45 (41.3) |  |
| Intracavitary fluid (%) |  |  | 1 |
| Yes | 117 (23.7) | 26 (23.9) |  |
| No | 376 (76.3) | 83 (76.1) |  |
| Hypertension (%) |  |  | 0.002 |
| Yes | 181 (36.7) | 58 (53.2) |  |
| No | 312 (63.3) | 51 (46.8) |  |
| Diabetes (%) |  |  | <0.001 |
| Yes | 115 (23.3) | 46 (42.2) |  |
| No | 378 (76.7) | 63 (57.8) |  |

**Table S2.** (continued)

| Characteristic | Non-AH/EC  (n=493) | AH/EC  (n=109) | P value |
| --- | --- | --- | --- |
| Obesity (%) |  |  | 1 |
| Yes | 100 (20.3) | 22 (20.2) |  |
| No | 393 (79.7) | 87 (79.8) |  |
| Family history of cancer (%) |  |  | 0.293 |
| Yes | 93 (18.9) | 26 (23.9) |  |
| No | 400 (81.1) | 83 (76.1) |  |
| History of abortion (%) |  |  | 0.63 |
| Yes | 193 (39.1) | 46 (42.2) |  |
| No | 300 (60.9) | 63 (57.8) |  |
| History of cesarean delivery (%) |  |  | 0.282 |
| Yes | 51 (10.3) | 7 (6.4) |  |
| No | 442 (89.7) | 102 (93.6) |  |
| CA125 (%) |  |  | <0.001 |
| >35 U/mL | 8 (1.6) | 23 (21.1) |  |
| ≤35 U/mL | 485 (98.4) | 86 (78.9) |  |
| CA19-9 (%) |  |  | <0.001 |
| >35 U/mL | 16 (3.2) | 28 (25.7) |  |
| ≤35 U/mL | 477 (96.8) | 81 (74.3) |  |
| Age (year) | 59.27±7.33 | 60.76±7.32 | 0.055 |
| Age at menarche (years) | 14.22±1.68 | 13.94±1.76 | 0.126 |
| Age at menopause (years) | 50.30±3.35 | 50.53±3.91 | 0.52 |
| Duration of menopause (years) | 8.97±7.40 | 10.23±7.58 | 0.111 |
| Gravidity | 2.86±1.64 | 2.72±1.22 | 0.4 |
| Parity | 2.04±1.05 | 2.04±0.94 | 0.942 |
| Height (cm) | 158.93±5.63 | 159.03±4.99 | 0.871 |
| Weight (kg) | 63.45±9.55 | 64.59±8.95 | 0.256 |
| BMI (kg/m²) | 25.10±3.53 | 25.52±3.25 | 0.259 |
| Fasting blood glucose (mmol/L) | 5.44±1.56 | 5.89±1.68 | 0.008 |
| Serum uric acid (μmol/L) | 272.17±64.76 | 304.44±84.03 | <0.001 |
| Hemoglobin (g/L) | 130.20±12.06 | 128.81±13.81 | 0.287 |
| Fibrinogen (g/L) | 2.74±0.58 | 2.90±0.70 | 0.015 |
| D-dimer (μg/mL) | 0.35±0.39 | 0.36±0.29 | 0.698 |

**Table S3.** List of abbreviations used in the study

| PMB | Postmenopausal bleeding |
| --- | --- |
| EC | Endometrial cancer |
| AH | Atypical hyperplasia |
| TVUS | Transvaginal ultrasound |
| ET | Endometrial thickness |
| IETA | International Endometrial Tumor Analysis |
| CA125 | Carbohydrate antigen 125 |
| CA19-9 | Carbohydrate antigen 19-9 |
| BMI | Body mass index |
| SVM | Support vector machine |
| GBM | Gradient boosting machine |
| NN | Neural network |
| RF | Random forest |
| XGBoost | Extreme gradient boosting |
| KNN | K-nearest neighbor |
| AdaBoost | Adaptive boosting |
| LightGBM | Light gradient boosting machine |
| AUC | Area under the receiver operating characteristic curve |
| F1 | F1-score |
| DCA | Decision curve analysis |
| P | P value |
| CI | Confidence interval |
| LASSO | Least absolute shrinkage and selection operator |
| LR | Logistic regression |
| SHAP | SHapley additive explanations |
| ACOG | American College of Obstetricians and Gynecologists |
| SOGC | Society of Obstetricians and Gynaecologists of Canada |
